# Purification and characterization of α-Amylase from Miswak *Salvadora persica*

**DOI:** 10.1186/1472-6882-14-119

**Published:** 2014-04-01

**Authors:** Saleh A Mohamed, Yaaser Q Almulaiky, Youssri M Ahmed, Omar AM Al-Bar, Ibrahim H Ibrahim

**Affiliations:** 1 Biochemistry Department, Faculty of Science, King Abdulaziz University, Jeddah 21589, Kingdom of Saudi Arabia; 2 Molecular Biology Department, National Research Center, Dokki, Cairo, Egypt; 3 Chemistry Department, Faculty of Science, Taiz University, Taiz, Yemen

**Keywords:** Miswak, α-Amylase, Purification, Characterization, Toothpaste

## Abstract

**Background:**

The miswak (*Salvadora persica*) is a natural toothbrush. It is well known that very little information has been reported on enzymes in miswak as medicinal plant. Recently, we study peroxidase in miswak. In the present study, the main goal of this work is to purify and characterize α-amylase from miswak. The second goal is to study the storage stability of α-amylase in toothpaste.

**Method:**

The purification method included chromatographaphy of miswak α-amylase on DEAE-Sepharose column and Sephacryl S-200 column. Molecular weight was determined by gel filtration and SDS-PAGE.

**Results:**

Five α-amylases A1, A4a, A4b, A5a and A5b from miswak were purified and they had molecular weights of 14, 74, 16, 30 and 20 kDa, respectively. α-Amylases had optimum pH from 6 to 8. Affinity of the substrates toward all enzymes was studied. Miswak α-amylases A1, A4a, A4b, A5a and A5b had Km values for starch and glycogen of 3.7, 3.7, 7.1, 0.52, 4.3 mg/ml and 5.95, 5.9 4.16, 6.3, 6.49 mg/ml, respectively. The optimum temperature for five enzymes ranged 40°C- 60°C. Miswak α-amylases were stable up to 40°C- 60°C after incubation for 30 min. Ca^+2^ activated all the miswak α-amylases, while Ni^2+^, Co^+2^ and Zn^+2^ activated or inhibited some of these enzymes. The metal chelators, EDTA, sodium citrate and sodium oxalate had inhibitory effects on miswak α-amylases. PMSF, *p*-HMB, DTNB and 1,10 phenanthroline caused inhibitory effect on α-amylases. The analysis of hydrolytic products after starch hydrolysis by miswak α-amylases on paper chromatography revealed that glucose, maltose, maltotriose and oligosaccharide were the major products. Crude miswak α-amylase in the toothpaste retained 55% of its original activity after 10 months of storage at room temperature.

**Conclusions:**

From these findings, α-amylases from miswak can be considered as beneficial enzymes for pharmaceuticals. Therefore, we study the storage stability of the crude α-amylase of miswak, which contained the five α-amylases, in toothpaste. The enzyme in the toothpaste retained 55% of its original activity after 10 months of storage at room temperature.

## Background

Chewing sticks are prepared from a variety of plant species and are customarily used for cleaning teeth in Asia, Africa, South America, and the Middle East [1]. The use of the chewing stick for cleaning teeth is an ancient custom which remains widespread in many parts of the world [2]. The World Health Organization has recommended and encouraged the use of chewing sticks as an effective tool for oral hygiene in areas where such use is customary [3]. *Salvadora persica* or Arak (miswak) (family name: Salvadoraceae)is the major source of material for chewing sticks in Saudi Arabia and much of the Middle East [4]. It has been shown that extracts of miswak posses various biological properties including significant antibacterial [5] and anti-fungal effects [6]. Almas [7] reported that miswak and chlorhexidine gluconate had the same effect on healthy human dentin. Chemical analysis of miswak has demonstrated the presence of glycosides, such as salvadoside and salvadoraside [8]; and flavonoids [9]. Silica acts as an abrasive and was found to help in removing stains from tooth surfaces [5,10]. Resins may form a layer on enamel that protects against dental caries [5]. Anti-microbial anionic components present in miswak include sulphate (SO_4_^2-^) and thiocyanate (SCN^**-**^**)**[11]. SCN^-^ acts as a substrate for salivary lactoperoxidase to generate hypothiocyanite (OSCN^-^) in the presence of hydrogen peroxide [12-14]. OSCN^-^ has been demonstrated to react with sulfhydryl groups in bacterial enzymes which in turn lead to bacterial death [11].

Amylases (EC 3.2.1.1) are a class of hydrolases widely distributed in microbes, plants and animals. They can specifically cleave the *O*-glycosidic bonds in starch, glycogen and several oligosaccharides [15]. α-Amylases and related amylolytic enzymes are among the most important enzymes and of great significance in the present day biotechnology. They could be potentially useful in the semisynthetic chemistry for the formation of oligosaccharides by transglycosylation [16]. The spectrum of amylase application has widened in many other fields, such as clinical, medicinal and analytical chemistry; as well as their widespread application in starch saccharification and in the textile, food, paper and pharmaceutical industries [17-20]. In plant, amylases also play a significant role in seed germination and maturation and are instrumental in starch digestion in animals resulting in the formation of sugars, which are subsequently used for various metabolic activities [21]. Amylases from different sources have been studied in great depth. For example, in germinating cereal grains, α-amylases are the most abundant starch-degrading enzymes. The enzymes are secreted by aleurone cells into the starchy endosperm where they degrade the starch grains [22].

It is well known that very little information has been reported on enzymes in miswak as medicinal plant. Recently, we study peroxidase in miswak [23]. Therefore, we will be studied some important enzyme in miswak such as α-amylase. In the present study, the main goal of this work is to purify and characterize α-amylase from miswak *S. persica* roots, as medicinal plant. The second goal is to study the storage stability of α-amylase in toothpaste.

## Methods

### Plant material

Miswak *Salvadora persica* L. (Salvadoraceae) root is wild plant and used as publicly available herbarium. Miswak root was purchased from local market of Jeddah, Kingdom of Saudi Arabia. Miswak was identified by Herbarium, Plant Division, Biology Department, King Abdulaziz University (voucher ID number 2215).

### Purification of miswak α-amylase

Five g of miswak peel were grinded in mortar with 20 mM Tris-HCl buffer, pH 7.2. The extract was filtered, centrifuged at 10,000 RCF for 15 min and dialyzed against 20 mM Tris-HCl buffer, pH 7.2. The supernatant was dialyzed against solid sucrose for concentrating the supernatant. The concentrated supernatant was used as crude extract. The crude extract was loaded on a DEAE- Sepharose column (10 × 1.6 cm i.d.) equilibrated with 20 mM Tris-HCl buffer, pH 7.2. The enzyme was eluted with a stepwise gradient from 0.0 to 0.4 M NaCl in the same buffer. Protein fractions exhibiting α-amylase activity were pooled in six peaks (A1 - A6). α-Amylase A1, A4 and A5 containing the highest activity were concentrated through dialysis against solid sucrose and separately loaded on Sephacryl S-200 column (90 × 1.6 cm i.d.) previously equilibrated with 20 mM Tris-HCl buffer, pH 7.2 and developed at a flow rate of 30 ml/h and 3 ml fractions were collected.

### α-Amylase assay

Amylase was assayed according to the procedure of Miller [24]. The reaction mixture was incubated at 37°C for 1 h in tubes containing 5 mg potato soluble starch, 50 mM Tris-HCl buffer, pH 7.2 and appropriately amount of enzyme solution and distilled water to give a final volume of 0.5 ml. The reaction was stopped by adding DNS reagent (0.5 ml), followed by incubation in a boiling water bath for 10 min followed by cooling. The absorbance was recorded at 560 nm. The enzymatically liberated reducing sugar was calculated from a standard curve using maltose. One unit of enzyme activity was defined as the amount of enzyme producing 1 μmol reducing sugar as maltose per hour under the standard assay conditions.

### Protein determination

Protein concentration was determined according to the dye binding method of Bradford [25] with bovine serum albumin as standard.

### Molecular weight determination

Molecular weight was determined by gel filtration technique using a Sephacryl S-200. The column was calibrated with cytochrome C (12.4 kDa), carbonic anhydrase (29 kDa), bovine albumin (66 kDa), alcohol dehydrogenase (150 kDa), β-amylase (200 kDa). Dextran blue (2,000 kDa) was used to determine the void volume (V_O_). The subunit molecular weight of the pure enzyme was determined by SDS-PAGE as described by Laemmli [26]. α- lactalbumin (14.4 kDa), soybean trypsin inhibitor (20 kDa), carbonic anhydrase (30 kDa), ovalbumin (43 kDa), bovine serum albumin (67 kDa) and phosphorylase b (94 kDa) were used as molecular weight standards for SDS-PAGE.

### Characterization of miswak α-amylase

#### Optimum pH

Miswak α-amylase activity was determined at various pH using different buffers, sodium acetate (pH 4.0-6.0) and Tris-HCl (6.5-9) at 50 mM concentration. The maximum activity was taken as 100% and % relative activity was plotted against different pH values.

### Km

The Km values were determined from Lineweaver-Burk plots by using starch and glycogen concentrations from 3-7 mg/ml.

### Optimum temperature

α-Amylase activity was determined at a temperature range of 20-80°C. The maximum activity was taken as 100% and % relative activity was plotted against different temperatures.

### Thermal stability

The enzyme was incubated at a temperature range of 20-80°C for 30 min prior to substrate addition. The % relative activity was plotted against different temperatures.

### Effect of metal ions

The enzyme was incubated with 2 mM solution of Ni^2+^, Ca^2+^, Co^2+^, Zn^2+^ Cu^2+^, pb^2+^ and Hg^2+^ for 30 min prior to substrate addition. The enzyme activity without metal ions was taken as 100% and % relative activity was determined in the presence of metal ions.

### Effect of metal chelators and inhibitors

α-Amylase activity was determined in the presence of metal chelators, EDTA, sodium citrate and sodium oxalate, 1,10 phenanthroline monohydrate and inhibitors phenylmethylsulfonyl fluoride (PMSF), p-hydroxymercuric benzoate (p-HMB) and dithiobis(2-nitrobenzoic acid)(DTNB) at a concentration of 2 mM. The enzyme activity without compound was taken as 100% and % relative activity was determined in the presence of compound.

### Paper chromatography

Twenty units of α-amylase (0.5 ml) was added to 1.25 ml of starch (2%), 0.625 ml of 200 mM Tris-HCl buffer, pH 7.2 and 0.125 ml distilled water. The mixture was incubated at 37°C overnight. Twenty μl of reaction mixture were applied to paper chromatography. Identification of the product of enzymatic reaction was proceeded by the descending paper chromatographic technique using paper whatman No.1 with solvent system n-butanol: acetic acid: water (180:45:75 ml) for a period of 24 h [27]. The chromatogram was dried then dipped in the alkaline silver oxide reagent [28]. This reagent was composed of two parts (1) 0.1 ml saturated aqueous silver nitrate in 100 ml acetone, and (2) 0.5 g NaOH dissolved in 5 ml water and diluted to 100 ml with ethanol. Part 1 was mixed immediately before use and a few drops of water were added, with stirring, until all the silver nitrate dissolved. The dried chromatogram was then dipped through the silver reagent end allowed to air dry 10 min to remove the acetone. It was then dipped through the ethanolic NaOH and again allowed to air dry. Spots begin to appear at once for most of saccharides, giving dark brown to black spots on a background which changed through yellow to brown. Glucose, maltose and maltotriose were used as standard.

### Preparation of toothpaste containing α-amylase

The toothpaste (Signal 2) product was prepared by mixing 5 g toothpaste with 100 units of miswak α-amylase crude extract.

### Determination of α-amylase activity in the toothpaste

α-Amylase activity of toothpaste product was determined by put 100 mg of toothpaste containing enzyme in test tube and measuring activity under standard assay conditions.

### Storage stability of the α-amylase in the toothpaste

The storage stability of the α-amylase in the toothpaste at room temperature was determined by monitoring the enzyme activity for ten months.

## Results and discussion

**Figure 1:**
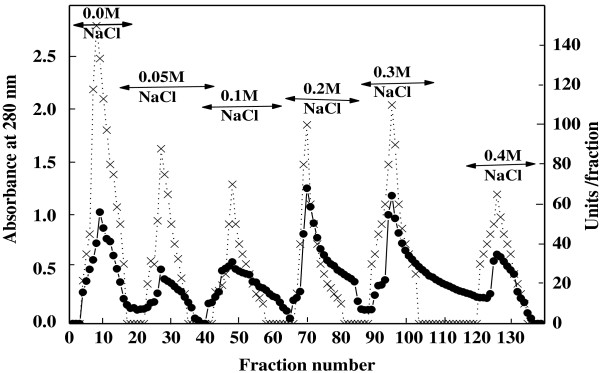
**A typical elution profile for the chromatography of miswak α-amylase on DEAE-Sepharose column (10 × 1.6 cm i.d.) previously equilibrated with 20 mM Tris-HCl buffer, pH 7.2 at a flow rate of 60 ml/h and 3 ml fractions.** Absorbant at 280 nm (•___• ), units/fraction (x ___ x).

**Figure 2:**
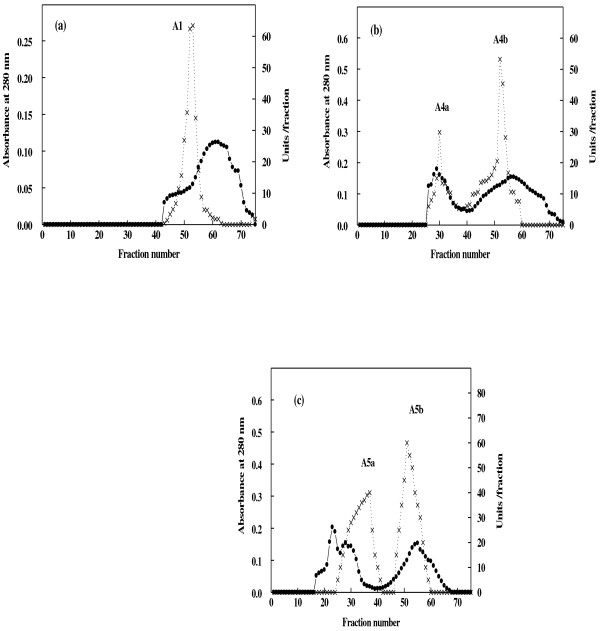
**Gel filtration of miswak α-amylases A1 (a), A4a and A4b (b) and A5a and A5b (c) DEAE-Sepharose fractions on Sephacryl S-200 column (90 × 1.6 cm i.d.).** The column was equilibrated with 20 mM Tris-HCl buffer, pH 7.2 at a flow rate of 30 ml/h and 3 ml fractions. Absorbant at 280 nm (•___• ), units/fraction (x ___ x).

**Figure 3:**
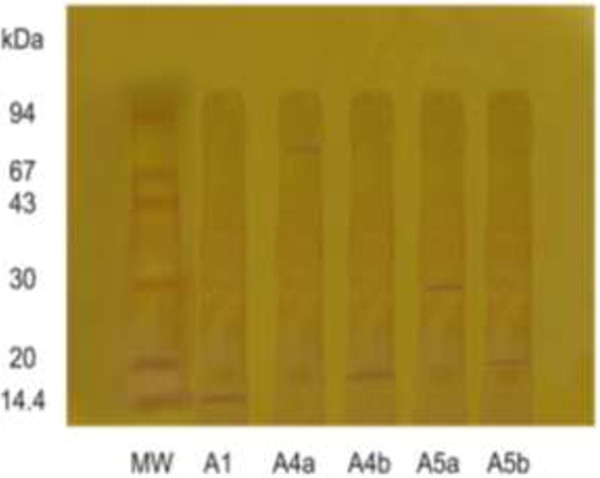
SDS-PAGE for homogeneity and molecular weight determination of miswak α-amylases A1, A4a, A4b, A5a and A5b.

The purification of α-amylase from miswak was summarized in Table 1. From the elution profile of the chromatography of α-amylase on DEAE–Sepharose, six peaks with α-amylase activity were detected (Figure 1), the negative adsorbed fraction and the fractions eluted with 0.05, 0.1, 0.2, 0.3 and 0.4 M sodium chloride and designated as miswak α-amylases A1, A2, A3 A4, A5 and A6. The miswak α-amylases A1, A4 and A5 with highest activity were separated on Sephacryl S-200 column to obtain α-amylases A1, A4a, A4b, A5a and A5b (Figure 2) with the highest specific activities 10210, 1385, 5571, 1500 and 10066 units/mg protein. The homogeneity of the purified A1, A4a, A4b, A5a and A5b were detected by SDS-PAGE (Figure 3), where each enzyme was appeared as one single protein band. The native molecular masses of miswak α-amylases A1, A4a, A4b, A5a and A5b were 14, 74, 16, 30 and 20 kDa, respectively, using Sephacryl S-200 column. These values were confirmed by SDS-PAGE and represented as a monomer (Figure 3). The molecular weights ranged from 20 to 57 kDa were detected for cereal amylases as wheat [29]**,** barley [30] and immature wheat [31]. For tuber *Pachyhizus erosus*[32]**,** ragi *Eleusine coracana*[33] α-amylases, their molecular weights were 40 and 45 kDa, respectively.

**Table 1:** Purification scheme for meswak α-amylase

**Step**	**Total protein (mg)**	Total activity (units)*	**Specific activity (units/mg protein)**	**Fold purification**	**Recovery %**
Crude extract	19.77	4908	248	1.0	100
Chromatography on DEAE- Sepharose					
0.0 M NaCl (A1)	1.83	1805.7	986.72	3.97	36.8
0.05 M NaCl (A2)	1.16	488.49	421.11	1.69	9.95
0.1 M NaCl (A3)	1.59	437.96	275.45	1.11	8.93
0.2M NaCl (A4)	2.48	647.62	261.14	1.05	8.92
0.3M NaCl (A5)	2.26	689.75	305.2	1.23	14.05
0.4 M NaCl (A6)	1.48	479.24	323.8	1.30	9.76
Gel filtration on Sephacryl S-200					
α-Amylase A1	0.1	1021	10210	41	20.8
α-Amylase A4a	0.12	163.3	1385	5.4	3
α-Amylase A4b	0.063	351	5571	22	7
α-Amylase A5a	0.2	300	1500	6	6.1
α-Amylase A5b	0.03	302	10066	40	6

*One unit of enzyme activity was defined as the amount of enzyme which liberated one μmol maltose per h under standard assay conditions.

**Figure 4:**
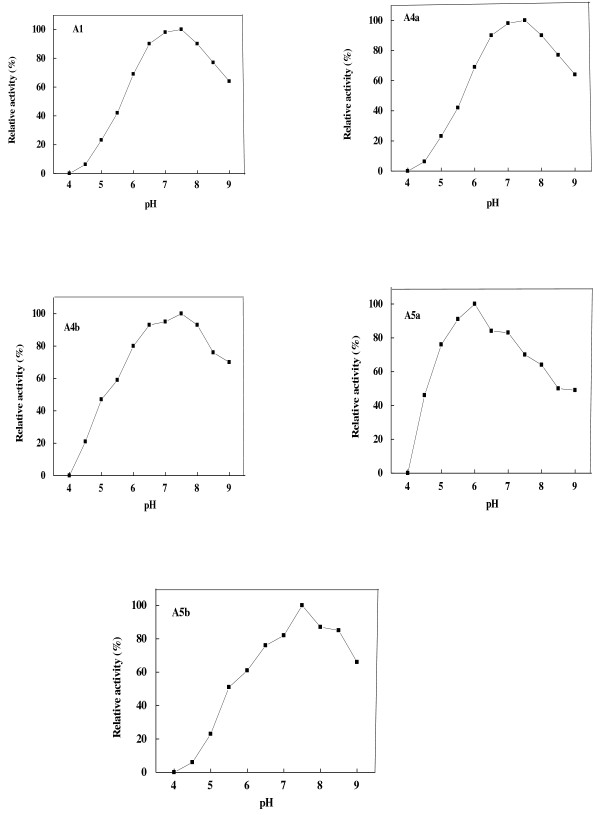
**Optimum pH of miswak α-amylases A1, A4a, A4b, A5a and A5b.** The reaction mixture contained in 0.5 ml: 2% starch, suitable amount of enzyme and 50 mM sodium acetate buffer (pH 4 - 6.0), 50 mM Trsi-HCl buffer (6.5 - 9). Each point represents the average of two experiments.

Different pHs were observed for miswak α-amylases (Figure 4). α-Amylase A5b was found to have pH optimum of 7.5. Similar pH optimum was observed for α-amylase from *P. erosus* tuber (pH 7.3) [32]. α-Amylases A4a and A5a were found to have pH optimum of 7-7.5 and 6.0, respectively. Acidic pH optima ranged from 4.5 to 5.6 were reported for α-amylases from wheat Sakha 69 [34], pea *Pisum sativum* seedlings [35], finger millet [33] and sorghum [36]. α-Amylases A1, A4b had broad optimum pH from 6.5 to 8. The broad range of pH optimum was reported for α-amylases from *Acremonium Sporosulcatum* (pH 6.8- 8.3) [37].

A number of carbohydrates were tested as substrates for miswak α-amylases A1, A4a, A4b, A5a and A5b (Table 2). All substrates tested had low activity to enzymes compared to starch. Glycogen, amylopectin and amylose had medium affinity toward these enzymes. The five enzymes had very low affinity toward α-cyclodextrin and β-cyclodextrin. These findings tend to suggest that the enzyme had high affinity toward high-molecular mass substrates containing α-1,4 and α-1,6 linkages. For tuber α-amylase, high-molecular-mass substrates containing the α-1,4-linkage were better substrates for the enzyme. The relative rate of hydrolysis of the polymeric substrate decreased with decreasing percentage of α-1,4-linkages and increasing percentage of α-1,6-linkages in the substrate, suggesting that the enzyme prefers high molecular-mass, amylose type material as the substrate. It hydrolyzed amylose at rates similar to those obtained with soluble starch, but it was considerably less active on amylopectin and showed no effect on maltose and maltotetraose [32]. It was observed that all the three amylases from finger millet were found to have a high affinity towards its natural substrates. The affinity for cereal starches were found to be in the order of ragi > rice > wheat > maize for α-1_(b)_ and α-3 whereas for α-2 it was in the order of ragi > wheat > rice > maize [33].

**Table 2:** Relative activities of miswak α-amylases toward different substrates

**Substrate**	**Relative activity %**				
	**A1**	**A4a**	**A4b**	**A5a**	**A5b**
Potato starch	100	100	100	100	100
Glycogen	71	55	49	60	61
Amylopectin	48	31	36	45	27
Amylose	20	15	25	48	11
α-Cyclodextrin	0.77	1.62	5	3	0.55
β-Cyclodextrin	0.07	0.18	0.29	0.53	0.07

The kinetic parameters of miswak α-amylases A1, A4a, A4b, A5a and A5b were studied on starch and glycogen as substrates. The Km values of the enzymes for hydrolyzing potato soluble starch (Km 3.7-5.2 mg/ml) and glycogen (4.6-6.49 mg/ml) are shown in Table 3. These values were similar to Km values reported for α-amylases from ragi *Eleusine coracana* (5.9-14.3 mg/ml starch) [33] and tuber *Pachyhizus erosus* (2.9 mg/ml starch) [32].

**Table 3:** Kinetic parameters of miswak α-amylases

**α -Amylase**	**Starch Km (mg/ml)**	**Glycogen Km (mg/ml)**
A1	3.7	5.95
A4a	3.7	5.9
A4b	4.1	4.6
A5a	5.2	6.3
A5b	4.3	6.49

**Figure 5:**
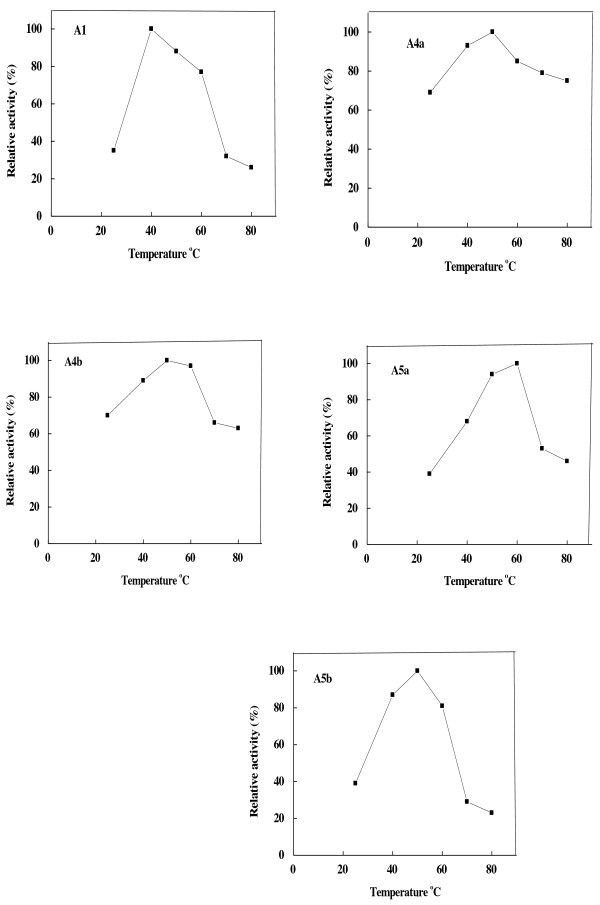
**Optimum temperature of miswak α-amylases A1, A4a, A4b, A5a and A5b.** The enzyme activity was measured at various temperatures using the standard assay method as previously described. Each point represents the average of two experiments.

**Figure 6:**
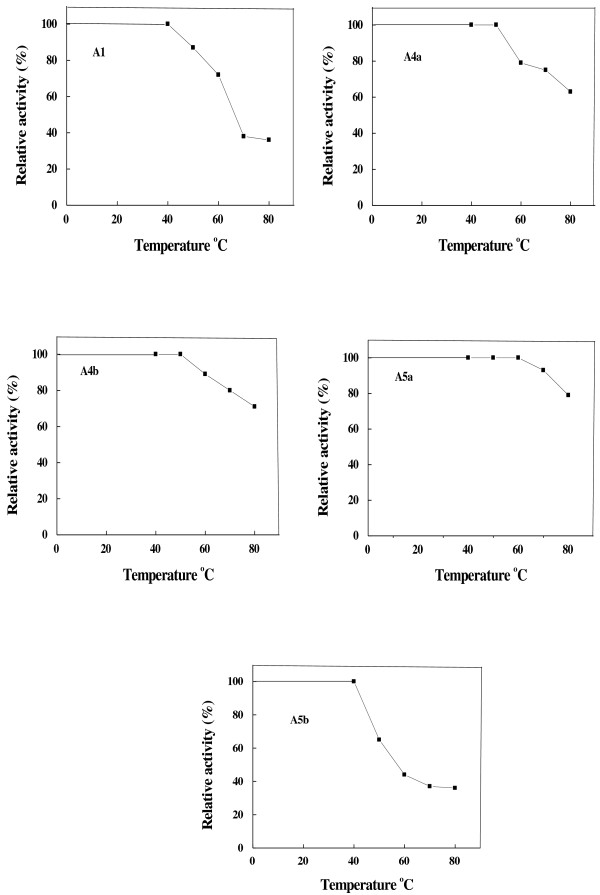
**Effect of temperature on the thermal stability of miswak α-amylases A1, A4a, A4b, A5a and A5b.** The reaction mixture contained in 0.5 ml: 50 mM Tris-HCl buffer, pH 7.5 and suitable amount of enzyme. The reaction mixture was preincubated at various temperatures for 30 min prior to substrate addition, followed by cooling in an ice bath. The enzyme activity was measured using the standard assay method as previously described. Activity at zero time was taken as 100% activity. Each point represents the average of two experiments.

α-Amylases A1, A4a, A4b, A5a and A5b had different temperature optima ranged from 40 to 60°C (Figure 5). These values resembled that of finger millet α-amylases α -1(b) and α -3 (45°C) and α -2 (50°C) [33]. Broad temperature optima (40-50°C) were detected in wheat α-amylase [29]. A temperature optimum at 37°C was reported for α-amylases from rice [38] and *P. erosus* tuber [32]. The miswk α-amylases were thermal stable up to 40-60°C (Figure 6). Similarly, α-amylase from wheat was stable up to 60°C [29]. On the contrary, wheat cv. Sakha 69 α-amylases AI and AII were thermally stable with half-maximal activity at 60 and 50°C, respectively [34].

The effect of metal ions showed that Ca^+2^ activated all the miswak α-amylases, while Ni^2+^, Co^+2^ and Zn^+2^ activated or inhibited some of these enzymes (Table 4). Cu^+2^ and Pb^+2^ caused partial inhibitory effect on all α-amylases, except of A4a and A4b with slightly activation. The complete loss of activity of the five enzymes was detected by Hg^2+^. The results indicated that A4a and A4b were more resistant toward metal ions than A1, A5a and A5b. We assumed that the inactivation by these metals may be due to their binding to either catalytic residues or by replacing the Ca^+2^ from the substrate binding site of the enzyme. Role of Ca^+2^ and Mg^+2^ in maintaining the stability and structure of the α-amylase is well documented [39]. Enhancement of amylase activity of Ca^+2^ ions is based on its ability to interact with negatively charged amino acid residues such as aspartic and glutamic acids, which resulted in stabilization as well as maintenance of enzyme conformation. In addition, calcium is known to have a role in substrate binding [40].

**Table 4:** Effect of metal cations on miswak α-amylases

**Metal**	**Relative activity %**				
	**A1**	**A4a**	**A4b**	**A5a**	**A5b**
Ni^2+^	43	205	131	103	40
Ca^2+^	108	152	141	110	195
Co^2+^	17	242	139	111	98
Zn^2+^	18	217	42	112	27
Cu^2+^	34	114	103	65	85
Pb^2+^	86	90	99	85	82
Hg^2+^	1	5	2	2	2

All the metal chelators are strong inhibitors of amylases as they are metalloenzymes. In cereal amylases Ca^2+^ is loosely bound to enzyme and can be removed by treating with metal chelators such as EDTA, EGTA, etc. [41]. EDTA has the same pattern of inhibition toward miswak α-amylases A1, A4a, A4b, A5a and A5b, where 2 mM of this chealator caused strong inhibitory effect (75-90% inhibition). In contrast, sodium citrate and sodium oxalate caused partial inhibitory effect (8-56% inhibition). For malted finger millet α-amylases, citric and oxalic acids were inhibiting these enzymes completely between 10 and 12.5 mM concentrations, respectively, and EDTA was found to be a competitive inhibitor of these enzymes at micro molar concentrations and inhibition was temperature dependent [42]. Further, amylase activities were almost completely abolished by 100 mM EDTA [32,43]. For the effect of inhibitors reagents such as PMSF, and 1,10 phenanthroline, they caused slight inhibitory effect on α-amylases A1, A4a and A4b and partial inhibitory effect on A5a and A5b. α-Amylases A1, A4a A5b were strongly inhibited by *p*-HMB, whereas A4b and A5a were partially inhibited. DTNB caused partial inhibitory effect on the enzymes A1, A4a, A4b and A5b, while it caused slight inhibitory effect on the enzyme A5a (Table 5). These results are similar to those reported for *G. sigillatus* α-amylase, where DNTB and *p*-CMB caused strong inhibition [44].

**Table 5:** Effect of metal chelating agents and inhibitors on miswak α-amylases

**Inhibitor**	**Relative activity %**				
	**A1**	**A4a**	**A4b**	**A5a**	**A5b**
EDTA	16	25	17	10	10
Sodium citrate	84	56	69	51	53
Sodium oxalate	92	61	44	53	60
PMSF	90	91	88	48	77
1,10 phenatroline	95	80	85	57	59
*p*-HMB	28	45	79	60	35
DTNB	66	65	65	91	47

**Figure 7:**
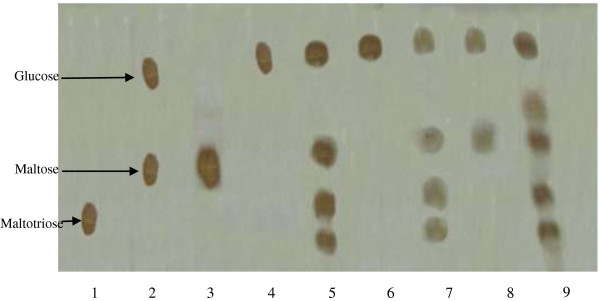
**Paper chromatography of the products of starch hydrolysis by miswak α-amylases.** 1- Authentic Maltotriose. 2- Mixture of Maltose and Glucose. 3- Authentic Maltose. 4- Authentic Glucose.5- Hydrolytic product of miswak α-amylase A1. 6- Hydrolytic product of miswak α-amylase A4a. 7- Hydrolytic product of miswak α-amylase A4b. 8- Hydrolytic Hydrolytic product of miswak α-amylase A5a. 9- Hydrolytic product of miswak α-amylase A5b.

The analysis of hydrolytic products after soluble starch hydrolysis by miswak amylases A1, A4a, A4b, A5a and A5b on paper chromatography revealed that glucose, maltose, maltotriose and oligosaccharide were the major product (Figure 7). On the basis of these major hydrolytic products, the three amylases A1, A4b and A5b are classified as the debranching enzymes (produced glucose, maltose, maltotriose and oligosaccharide). A4a is classified as the exoamylase (produced glucose) and A5a is classified as the endoamylase (produced glucose and maltose). These results are similar to those reported for α-amylase from *P. Americana*[45]**,***Bacillus claussi* LT21 [46] and *Pichia burtonii*[47].

The crude α-amylase of miswak in the toothpaste was stored at room temperature and measured its activity at intervals of months. As shown in Table 6 the α-amylase in the toothpaste product retained 55% of its original activity after 10 months.

**Table 6:** Storage stability of the crude α-amylase from miswak in the toothpaste

**Month**	**% relative activity**
1	100
2	99
3	91
4	85
5	70
6	71
7	69
8	62
9	59
10	55

## Conclusions

We purified five α-amylases from miswak, which characterized by: (i) pH optima ranged from 6.0 to 7.5, (ii) broad substrate specificity with starch analogs, (iii) thermalstable enzyme activity ranged from 40 to 60°C, (iv) high tolerance towards some of metal ions, (v) debranching enzymes, exoamylase and endoamylases. From these findings, α-amylases from miswak can be considered a beneficial enzyme for pharmaceuticals. Therefore, we study the storage stability of the crude α-amylase of miswak, which contained the five α-amylases, in toothpaste, where the enzyme in the toothpaste retained 55% of its original activity after 10 months at room temperature.

## Competing interests

The authors declare that they have no competing interests.

## Authors’ contributions

MS, MY, AY, Al-O and II performed all experiments and read and approved the final manuscript.

## Pre-publication history

The pre-publication history for this paper can be accessed here:

http://www.biomedcentral.com/1472-6882/14/119/prepub
